# Investigating the synchronization of hippocampal neural network in response to acute nicotine exposure

**DOI:** 10.1186/1743-0003-7-31

**Published:** 2010-07-13

**Authors:** David Akkurt, Yasemin M Akay, Metin Akay

**Affiliations:** 1 Harrington Department of Bioengineering, Fulton School of Engineering ASU, Tempe AZ, USA; 2 Department of Biomedical Engineering, Cullen College of Engineering, University of Houston, Houston TX, USA

## Abstract

Previous studies suggested that γ oscillations in the brain are associated with higher order cognitive function including selective visual attention, motor task planning, sensory perception, working memory and dreaming REM sleep. These oscillations are mainly observed in cortical regions and also occur in neocortical and subcortical areas and the hippocampus. In this paper, we investigate the influence of acute exposure to nicotine on the complexity of hippocampal γ oscillations.

Using the approximate entropy method, the influence of acute nicotine exposure on the hippocampal γ oscillations was investigated. The hippocampal γ oscillations have been generated in response to the 100 Hz stimulus and isolated using the visual inspection and spectral analysis method. Our central hypothesis is that acute exposure to nicotine significantly reduces the complexity of hippocampal γ oscillations. We used brain-slice recordings and the approximate entropy method to test this hypothesis. The approximate entropy (complexity) values of the hippocampal γ oscillations are estimated from the 14 hippocampal slices. Our results show that it takes at least 100 msec to see any hippocampal activities in response to the 100 Hz stimulus. These patterns noticeably changed after 100 msec until 300 msec after the stimulus Finally, they were less prominent after 300 msec. We have analyzed the isolated hippocampal γ oscillations (between 150 and 250 msec after the stimulus) using the approximate entropy (ApEn) method. Our results showed that the ApEn (complexity) values of hippocampal γ oscillations during nicotine exposure were reduced compared to those of hippocampal γ oscillations during control, and washout. This reduction was much more significant in response to acute nicotine exposure (p < 0.05) compared to those during control and washout conditions. These results suggest that the neural firing becomes regular and the hippocampal networks become synchronized in response to nicotine exposure.

## Introduction

Nicotine, C_10_H_14_N_2_, a tertiary amine compound, is a very toxic, light yellow alkaloid which is produced in tobacco plants as a result of the leaves' being damaged [1,2]. The roots of the damaged plants synthesize nicotine as a reaction to hormones released by the damaged tissue and then transport it to the leaves. Nicotine is stored in concentrations of 2-8 percent by weight in the leaves which becomes the primary psychoactive ingredient in tobacco smoke. It is very soluble in water and nonpolar solvents. It is absorbed rapidly by the body, either through the skin or smoking, crosses the blood-brain barrier and stimulates nicotinic-cholinergic receptors of the CNS, causing an increase in heart rate, blood pressure and some cognitive functions in humans and experimental animals [3].

Nicotine exerts its effects through the activation of nicotinic acetylcholine receptors (nAChRs) in the multiple areas of the brain such as hippocampus, amygdala and prefrontal cortex (PFC) [4]. These receptors are ion channels and their locations are very important as far as the understanding of their physiological impact on neuronal activity is concerned [4,5]. A series of studies have pointed out the crucial role these areas play in working memory function. Particularly, stimulation of the (nACHRs) in the hippocampus was proven critical for optimal memory performance [5,6].

The mounting evidence suggests that the synaptic plasticity, such as long-term potentiation (LTP), takes part during the learning and memory process. LTP has been investigated in detail in the Shaffer collateral CA1 region of the hippocampus where nicotine enhances LTP induction and modulates the synaptic plasticity--thereby playing a crucial role on attention performance [7,8].

It has been proposed that higher cognitive functions, such as learning, memory, attention and exploratory behavior, could be represented in the CA1 region of the hippocampal area of the brain by a distributed neuronal network synchronization on an oscillatory mode as the gamma-band or 40 Hz (30-80 Hz) oscillations [9]. Gamma oscillations appear primarily in the neocortex, hippocampus, thalamus and related structures. In the hippocampus, they are generated by the entrainment of populations of mutually interconnected interneurons and can be stimulated by brief high-frequency tetanic stimuli to the Schaffer collateral CA1 pathway in hippocampal slice preparations [9-11].

There is a strong link between nicotine administration, neuronal nicotinic systems and higher cognitive functions evident from many studies in rodents, primates, zebrafish and humans [12-15]. Fries et al suggested that nicotine plays an important role in regulating beta oscillations which are associated with higher cognitive functions. Gray et al suggested that nicotine influences cognition by enhancing synaptic transmission. Radial-arm maze is an important tool for the testing of the working memory in rats and it has shown a significant improvement in this memory type with both acute and chronic nicotine treatments [14-16]. However, nicotine-induced improvement in attention in rats seemed to be strain-dependent. Sprague-Dawley rats showed better performance than the Lister hooded rats in attention tests, which suggested that there may be crucial, strain-related differences in the mechanisms of the nicotinic receptors--or neuronal systems related to higher cognitive functions--and nicotine reinforcement [17,18].

In non-smoking adults with ADHD (attention deficit hyperactivity disorder), nicotine seemed to improve attention [19]. In Alzheimer's Disease (AD), which is characterized by loss of cognitive functioning, there is strong evidence that nicotinic receptors play an important role in the pathogenesis of the disorder. Postmortem evaluations of the brains of AD patients have shown a substantial loss in nicotinic receptors in cortex and striatum. According to the growing amount of findings, accumulation of amyloid plaques--which are the baseline indicator in the brain for AD--is interfered by nicotine, which is very promising for the future of AD patients [20,21].

In many studies on the effect of nicotine in Schizophrenic patients, despite many positive findings for attention and spatial processing, a majority of the tests related to the measurements of memory functioning gave negative results [22]. An important body of evidence suggests that, nicotinic receptors play a role in the pathophysiology of Parkinson's Disease (PD), revealing that smoking may provide some protection against the onset of PD. However, a wider rage of intense studies needs to be performed before drawing any significant conclusions [23].

There are some studies indicating nicotine's positive effects in improving cognitive functioning in individuals with Tourette's and Down's syndromes. In spite of promising results, these studies have been limited and more thorough research is needed in these cases [24]. In the current study, we have investigated the effect of nicotine on the complexity of the neurons and the activity of the gamma oscillations in the Schaffer CA1 cell line on the hippocampal slices.

## Methods

### Preparation of slices

All experimental protocols were approved by the Institutional Animal Care and Use Committee of Arizona State University; Furthermore, all experiments were carried out in accordance to such protocols. In this experiment, Sprague Dawley rats age 23-36 days old were anesthetized by isoflurane then immediately decapitated. Without delay the brain was removed and placed in chilled (4°C) artificial cerebrospinal fluid (ACSF). The ACSF solution consisted of the following 135 mM NaCl, 3 mM KCl, 16 mM NaHCO_3_, 1 mM MgCl, 1.25 mM NaH_2_PO_4_, 2 mM CaCl_2_, and 10 mM Glucose [25]. The above solution was continuously bubbled with carbogen (95-5% Oxygen - Carbon Dioxide gas mixture). This solution was chosen because of its success in prior work as described in [25]. The Brain tissue was cold sliced using a vibratome (Vibratome 1000, Vibratome Co., St. Louis, MO, USA) while immersed in chilled ACSF bulled with carbogen. Coronal sections at 400 μm of the chilled brain tissue were made using the vibratome followed by transverse sectioning to create tissue containing hippocampus and entorhinal cortex.

All Sectioning was done while the tissue was under the protection of the chilled ASCF. The cut slices were immediately transferred to a incubation chamber containing ACSF, where the atmosphere was 95% Oxygen and 5% Carbon Dioxide. The Slices were incubated in this chamber at 24°C for 1 hour prior to recording. After the incubation period a slice was transferred to a liquid-air interface chamber to begin recording (Fine Science Tools Inc., Foster City, CA, USA). The slice was suspended on a nylon mesh in the recording chamber where ACSF bubbled with carbogen continually flowed past the slice at a rate of 2-2.5 ml/min. Recording chamber temperature was closely monitored and regulated by a feedback circuit set at 34 ± .3°C. The slice was allowed 15 minutes of rest before any stimulation was made. In this experiments we used (-) nicotine (Sigma Chemical Co., St. Louis, MO).

### Electrophysiology

Using borosilicate glass electrodes (borosilicate micropipettes, WPI, Sarasota, FL) with a pulled tip of about 1 μm, extracellular field potential recordings were made on the stratum pyramidale of the CA1 cell layer. The micropipette was filled with a standard 2 M NaCl solution [25]. The slices were stimulated with double intensity (2 times threshold, 2 T) which is approximately between 8 V and 14 V. Double intensity (2 T) was chosen due to its ability to induce γ oscillations. Once the stimulation voltage was set for a particular slice it was not changed for the remainder of the experiments on that slice. The stimulating voltage was delivered via bipolar twisted platinum wire electrode on the Schaffer collaterals. In order to induce tetanus, the stimulation signal is changed from a single pulse lasting a 100 μ second to a train of 100 Hz signal lasting 200 ms delivered by a model 2100 A-M Systems Isolated Pulse Stimulator (Carlsborg, WA) [25-30]. The tetanic stimulation level is determined by the threshold level voltage that must be applied to the Schaffer collaterals to elicit a response. In order to induce γ oscillations, the stimulation intensity needs to be set at above threshold and double intensity stimulation was used. Signals were recorded using an Axoclamp 900A (Axon Instruments, Inc., Union City, CA, USA) amplifier. Prior to each drug experiment a single stimulus (8-14 V, 100 μs) was delivered to the slice to insure the slice was viable and the recording and stimulating electrodes were in the optimum positions to record synaptic transmission. Once the optimum electrode placement was determined, signal stability was insured by recording anywhere from 20-60 min prior to actual nicotine administration. After the recordings were stabilized the chemical was started and tetanic stimulations were applied every 10 minutes. The concentration of the nicotine used in this experiment was 100 μ Molar. Only a single concentration was used because previously it was shown by Song et al. that 100 μ Molar was the minimum dose that would cause tetanic beta oscillations. Nicotine was dissolved in ACSF and delivered to the slice at the same rate as standard ACSF solution without drug [27-30].

### Data acquisition and analysis

All signals were recorded with pClamp 10.2 via Axon Digidata 1440A (Axon Instruments, Inc., Union City, CA, USA). The recording parameters were set such that the sampling frequency was 100 kHz and low pass filtered with a cut off at 1 kHz. All signals were then transferred to a PC for later analysis. Offline analysis was done on Matlab using various tools such as approximate entropy and Matching Pursuit techniques.

### Approximate entropy

Quantitative changes in the complexity of a signal are traditionally quantified using tools such as nonlinear dynamical system analysis methods. These traditional complexity measures work well when signals are long in duration as they are length dependent[31-33]. For biological signals with short signal durations of 100-5000 points, these traditional complexity measures that measure signal irregularity don't work well. A statistically efficient measure to quantify the irregularity of a signal has been formulated to overcome these shortcomings of these previous complexity measures, commonly known as approximate entropy [31-33].

To analyze the field potential recordings generated by the hippocampal CA1 layer approximate entropy (ApEn) was ultimately used. ApEn is a statistical measure that both smoothens the transient interference and suppresses the influence of noise by properly adjusting the algorithms parameters. This said irregularity measure can be applied both to deterministic and stochastic signals [33,34]. Considering that the outputs of a biological systems can be rather complex and could be either deterministic or stochastic or both, thus it is crucial that this complexity measure be able to handle both signal types. The algorithm summarizes a time series into a non-negative number, with higher values representing more irregular systems [34].

Approximate entropy is calculated by taking segments *X*(*i*) through *X*(*N - m + *1) defined by *X*(*i*) = [*x*(*i*), ..., *x*(*i *+ *m *- 1)]. The difference between *X*(*i*) and *X*(*j*), *d*[*X*(*i*), *X*(*j*)] as the maximum absolute difference between their related scalar elements can be estimated as:

(1) $$d\left[X(i),X(j)\right]=\max_{k=0,m-1}\left[\left|x(i+k)-x(j+k)\right|\right]\leq r$$

assuming that all the differences between the corresponding elements will be less than the threshold *r*.

For any given *X*(*i*), the ratio of the difference between *X*(*i*) and *X*(*j*) smaller than the threshold *r *to the total number of vectors (*N - m +*1) is obtained as:

(2) $$\begin{array}{ccc} C_{r}^{m}(i)=N_{r}^{m}(i)/(N-m+1) & for & i=1,...,N-m+1 \end{array}$$

The approximate entropy, ApEn(*m*, *r*), can be estimated as a function of the parameters *m *and *r *as follows:

(3) $$\text{ApEn}(m,r)=\underset{N\to \infty }{\lim}\left[\Phi^{m}(r)-\Phi^{m+1}(r)\right]$$

where

(4) $$\Phi^{m}(r)=\sum_{i=1}^{N-m+1}\ln C_{r}^{m}(i)/(N-m+1)$$

In practice, the approximate entropy values can be estimated for a signal with *N *samples as:

(5) $$\text{ApEn}(m,r,N)=\left[\Phi^{m}(r)\Phi {-}^{m+1}(r)\right]$$

The parameter *m *is the embedding dimension of the analyzed signals and the parameter *r *is the threshold to suppress the noise in the signal. Throughout this study we have chosen *m *= 2 as described in previous works [34]. The parameter *r *can be chosen as 0.1 SD(*x*(*i*)), where SD(*x*(*i*)) represents the standard deviation of the original signal *x*(*i*).

## Results

**Figure 1:**
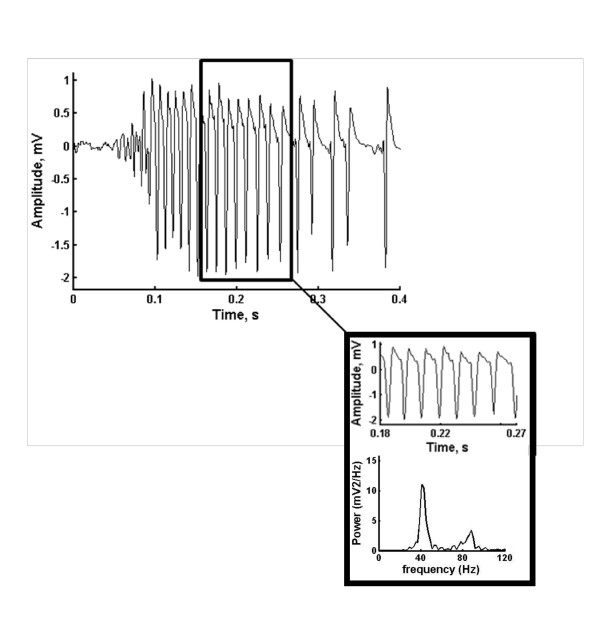
Shows tracings of a typical hippocampal oscillation signal (upper panel), the isolated hippocampal γ oscillation segment between 150 msec and 250 msec after the stimulus (middle panel) and the corresponding spectral components of the isolated hippocampal γ oscillation segment (lower panel).

Before the analysis of hippocampal field potential recordings using ApEn, all samples were initially cut by removing the stimulation artifact. All signals were then sampled at 2 kHz. Moreover, the data was detrended using piecewise linear trend removal and removing their respective linear trends to produce an overall detrended signal. All data was band pass filtered at (30-300) Hz. Figure 1 shows tracings of a typical hippocampal oscillation signal. Please note that it takes at least 100 msec to see any hippocampal activities in response to the 100 Hz stimulus. The patterns of hippocampal γ oscillations were irregular and random at least 100 msec after the stimulus. But, these patterns noticeably changed between 100 msec and 300 msec and became more regular. Finally, the late (300-500 msec) segment, the patterns became more irregular and random. We isolated the γ oscillation segments (between 150 msec and 250 msec after the stimulus) by the visual inspection (Figure 1, middle panel) and by using the spectral components of hippocampal oscillations in the visually isolated segment (Figure 1, lower panel) as a reference. Note that the main spectral component was around 60 Hz which is considered to be in the hippocampal gamma oscillation range (30-60 Hz). These isolated hippocampal γ oscillations were analyzed using the ApEn method as detailed above.

**Figure 2:**
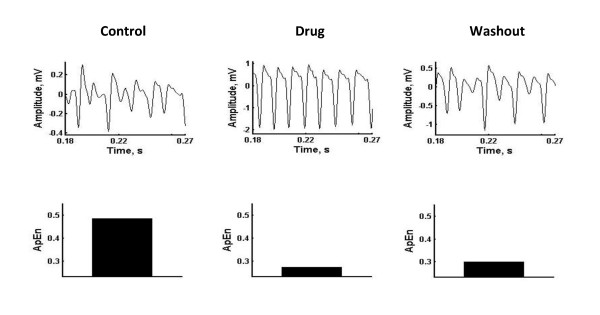
S**hows tracings of isolated hippocampal γ oscillations in response to the 100 Hz before (control), during nicotine exposure and after acute nicotine injection (washout) in the upper panel and the corresponding complexity values of these isolated hippocampal γ oscillations in the lower panel.**

Figure 2 shows tracings of isolated hippocampal γ oscillations in response to the 100 Hz before (control), during nicotine exposure and after acute nicotine injection (washout) in the upper panel. The corresponding complexity (approximate entropy) values of these hippocampal oscillations were 0.49, 0.27, and 0.30 respectively, as shown in the lower panel in Figure 2. The complexity value was reduced during nicotine exposure, suggesting the emergence of strong synchronization and regular firing. During washout period, the complexity value was increased again by suggesting more irregular pattern like before nicotine exposure.

**Figure 3:**
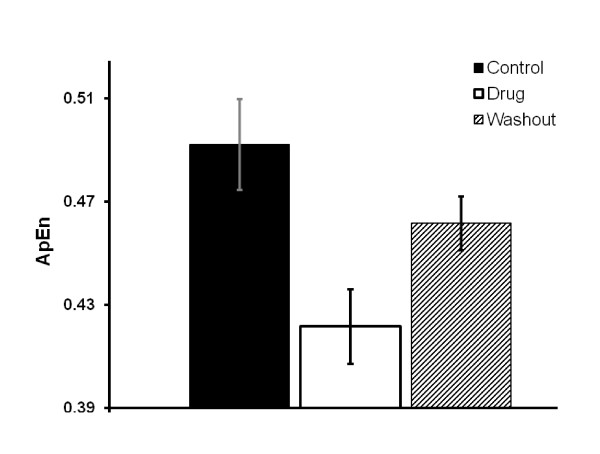
Shows the mean complexity values of the hippocampal oscillations during control, nicotine exposure and washout

Figure 3 shows the mean complexity values of the hippocampal oscillations during control, nicotine exposure and washout where n = 14. These values were 0.49 ± 0.01 before (control), 0.42 ± 0.02 during nicotine exposure, and 0.46 ± 0.02 after acute exposure respectively.

Our results suggest that the complexity values the hippocampal oscillations during control were more or less the same and high during washout periods. However, the mean complexity value of the hippocampal oscillations in response to nicotine exposure were reduced compared to those of control and washout periods (p < 0.05). These results furthermore suggest that the hippocampal γ oscillations in response to nicotine exposure are unique and indicate the emergence of more synchronization of the hippocampal neural networks since hippocampal neural firings become regular and deterministic processes in response to the 100 Hz stimulus.

## Discussion and conclusion

It has been widely reported that γ oscillations in the brain are associated with higher order cognitive function including selective visual attention, motor task planning, sensory perception, working memory and dreaming REM sleep. These oscillations are mainly observed in cortical regions and also occur in neocortical and subcortical areas and the hippocampus. Hippocampal neurons play a critical role in information processing and decision making.

Although many components of tobacco smoke are harmful to the brain, and cardiorespiratory systems, nicotine has been found to be useful for the improvement of cognitive functions including working memory and executive function. Furthermore, it has been found to be neuroprotective [35]. It has been widely reported that acute exposure to nicotine improves vigilance, selective attention, memory, and executive function in human and animals. The activation of the cholinergic system and subsequent downstream effects on neurotransmitter release including dopamine and serotonin are responsible for this [35].

In addition, the nicotine was also proposed for the treatment of Alzheimer's disease (AD) and schizophrenia. The nicotinic-cholinergic system was suggested as a novel therapeutic mechanism for treatment in schizophrenia. Several cholinergic receptor agonists were proposed to characterize central nervous system cholinergic function and as potential candidates for the treatment of schizophrenia as well as dementia of the Alzheimer's type. In addition, the alpha7 nicotinic receptor agonists have also been found to be effective on learning and memory in animals. Thus, these alpha7 nicotinic receptor agonists appear to be excellent candidates for the treatment of impaired sensory gating, cognitive deficits and negative symptoms in people with schizophrenia [36-39].

Martin et al discussed a novel model of nicotinic-cholinergic dysfunction which may be responsible for the impairment of sensory gating in schizophrenia and the related neurobiological and genetic mechanisms as well as the treatment of this disease with the use of nicotine, clozapine and the potential place of the new alpha7 nicotinic receptor agonists [39,40].

In this paper, we focus only on the influence of nicotine exposure on the complexity of hippocampal γ oscillations. We used a nonlinear dynamics approach for data analysis since the γ oscillations reflect the integrated properties of the underlying dynamics of the hippocampal neural network and therefore exhibit highly complex/irregular features. Specifically, the approximate entropy is a computationally efficient complexity analysis method that is able to produce accurate estimations of the complexity of the biological signals complexity (irregularity). Our study suggests that that the sequence of 100 Hz stimulations triggered a response consisting of γ oscillations (30-120 Hz) 150 msec after the application of the stimulus. The most striking observation was the reduction of complexity values of isolated hippocampal γ oscillations in response to acute nicotine exposure. It furthermore suggests the strong synchronization of hippocampal neural network in the mid phase (gamma oscillation segment) in response to acute nicotine exposure. But, the reorganization of the hippocampal network was a reversible process since all the complexity values were restored after the washout period.

## Competing interests

The authors declare that they have no competing interests.

## Authors' contributions

All the authors contributed equally to this work and have read and approved the final manuscript.
